# The incidence and influence of the donor corneas positive for herpesviridae DNA in keratoplasty

**DOI:** 10.1007/s00417-020-04984-2

**Published:** 2020-10-24

**Authors:** Jing-hao Qu, Rong-mei Peng, Ge-ge Xiao, Hong-qiang Qu, Ting Yu, Shuang Zhang, Jing Hong

**Affiliations:** 1 Department of Ophthalmology, Peking University Third Hospital, Beijing, China; 2 Beijing Key Laboratory of Restoration of Damaged Ocular Nerve, Peking University Third Hospital, Beijing, China

**Keywords:** Donor cornea, HSV-1, HSV-2, VZV, CMV, EBV, Transplantation, RT-PCR

## Abstract

**Purpose:**

We detected the DNA of herpes simplex virus type 1 (HSV-1), herpes simplex virus type 2 (HSV-2), varicella-zoster virus (VZV), cytomegalovirus (CMV), and Epstein-Barr virus (EBV) in donor corneas and assessed the clinical outcomes of recipients who received virus-positive grafts.

**Method:**

All donor corneas were analyzed for the presence of HSV-1, HSV-2, VZV, CMV, and EBV by real-time PCR from April 2017 to July 2019. The medical records of the transplant patients who received virus-positive grafts were reviewed.

**Result:**

Twenty-three (2.44%) donor cornea buttons tested positive for herpesviridae DNA. The positivity rates of HSV-1, CMV, VZV, and EBV were 0.74%, 0.85%, 0.64%, and 0.21%, respectively.

**Conclusion:**

We suggest that the corneas from donors who had cancer, donors who were inpatients, and donors who had immunodeficiency or who were on immunosuppressive therapy should be tested for herpesviridae DNA before transplantation. Finally, HSV-1 can be transmitted from graft to recipient, but that CMV cannot be transmitted according to our observations. The donor corneas found to be HSV-1-positive have to be discarded and not used for keratoplasty.

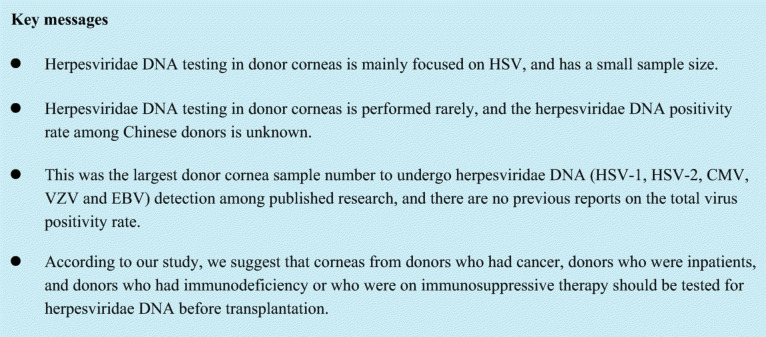

## Introduction

The cornea is composed of avascular tissue, making cornea transplantation the most successful human tissue transplantation procedure. With improvements in science and technology, including updated eye banking procedures, operating techniques, immunosuppressive, and antiviral drugs [1], the success rate of total corneal transplantation is more than 90% at 1 year and 70% at 5 years [2]. In recent years, researchers have implicated a formerly unknown cause of corneal graft edema, herpes simplex virus (HSV) infection in some cases of primary graft failure [3–5]. This suggests that virus infection is a reason for graft failure in corneal transplantation. Most recipients have no history of virus infection; therefore, we wondered whether the virus already existed in the recipients or was transmitted from the donor cornea to the recipient.

The donor corneas and preservation fluid are tested for bacterial and fungal pathogens, as they are believed to have direct impacts on the quality of grafts and the safety of recipients. Furthermore, the cornea donors are also tested for hepatitis B virus (HBV), hepatitis C virus (HCV), syphilis, and human immunodeficiency virus (HIV) before organ donation. Virus detection is not a routine examination in most eye banks. Surprisingly, data on herpesviridae DNA positivity rates in the donor corneas and the risk of transmission to recipients are scarce [6].

There are a few cases of graft-to-recipient transmission of viruses reported in the literature [3, 7, 8]. In 2001, HSV-1 DNA was isolated from a donor cornea, and the transmission of HSV-1 through transplantation was confirmed by genetic characterization [9]. Subsequently, a prospective study detected HSV DNA in the corneal buttons of 38 corneal graft recipients and in donor scleral remnants. Two donor corneas were found to be positive for HSV-1 DNA, and endothelial transplantation failure occurred in one of the recipients 4 months later [10].

Moreover, herpesviridae DNA testing in donor corneas is rarely performed, and the herpesviridae DNA positivity rate among Chinese donors is unknown. All the existing studies focus on HSV-1, which can be transmitted from graft to recipient, and there are no reports in the literature focusing on other members of herpesviridae, including varicella-zoster virus (VZV), cytomegalovirus (CMV), and Epstein-Barr virus (EBV). The purpose of this study was to verify the herpesviridae DNA positivity rate among Chinese patients and how virus-positive grafts influence corneal transplantation.

## Materials and methods

### Donor corneas

The information of 569 donors was registered on the unified organ donation website from April 2017 to July 2019, and informed consent was obtained from all participants and/or their legal guardians. Tests for HBV, HCV, syphilis, and HIV were all negative before organ donation. Under sterile conditions, a suitable trephine was used to strip a 3- to 4-mm section of the sclera and posterior cornea within 12 h after donor death. Then, corneoscleral buttons were obtained and stored in Optisol-GS (Bausch & Lomb, Irvine, CA, USA) at 4 °C. The endothelial cell density (ECD) of all donor corneas was quantified by a certified technician at our eye bank using an EB-3000 XYZ eye bank specular microscope (HAI Laboratories Inc., Lexington, MA, USA). The study was performed according to the tenets of the Declaration of Helsinki and was approved by the local ethics committee.

### Surgical technique and sampling

In this study, all procedures were performed by an experienced surgeon (Jing Hong) at Peking University Third Hospital. The donor circular limbal corneas comprising the corneal endothelium were obtained during surgery with aseptic precautions and immediately delivered to the laboratory for virological analyses. The samples were obtained during consecutive cornea transplant procedures. All samples were tested for HSV-1, HSV-2, VZV, CMV, and EBV DNA.

### Herpesviridae DNA isolation

We extracted DNA from the corneal tissues using a QIAamp DNA Mini Kit (Qiagen, Hilden, Germany) according to the manufacturer’s instructions. In brief, the corneal rims were cut into small pieces, placed in a 1.5-mL microcentrifuge tube, and digested with ATL buffer and proteinase K. The extracted DNA was diluted in water; a total of 50 ng was subjected to PCR.

### Herpesviridae DNA detection

HSV-1, HSV-2, VZV, CMV, and EBV were detected using qualitative commercial, TaqMan-based methods (HSV-1/HSV-2 Typing Real-Time PCR Kit Z-SD-0136-02, VZV Real-Time PCR Kit OD-0024-02, CMV Real-Time PCR Kit Z-OD-002-02, and EBV Real-Time PCR Kit Z-OD-0023-02, Liferiver Bio–Tech Corp., China), in accordance with the manufacturer’s instructions. Real-time PCR assays were performed using reagents from PE Biosystems (PE Applied Biosystems, Foster City, CA). The limit of detection for all herpesviridae DNA was 10 copies/μg. Each sample was processed with the addition of an internal control to assess isolation and amplification efficacy. Positive and negative controls, as well as internal controls, were provided by the kit manufacturers.

### Postoperative follow-up

The clinical outcome of transplantation was assessed by best-corrected visual acuity (BCVA), intraocular pressure (IOP), ECD, and complications at 1 and 3 months postoperatively. The endothelial cell (EC) loss rate was calculated according to the ECD. The ECD of the central area was measured by in vivo confocal microscopy (IVCM; HRT III: Heidelberg Engineering, Heidelberg, Germany). Graft attachment was assessed with anterior segment optical coherence tomography (AS-OCT; Carl Zeiss Meditec, Dublin, CA, USA). The same certified ophthalmic technician performed all postoperative tests in patients using the same microscope.

## Statistical analysis

Statistical analysis was performed with SPSS 18.0 (SPSS, Inc., Chicago, IL, USA). Donor age, ECD, time from death to preservation, and day of death to day of surgery in the DNA-positive and DNA-negative groups were compared using independent-samples *T* tests. Donor sex, cause of donor death, and inpatient assessment were evaluated using the chi-square test. The EC loss in recipients who received the virus-positive corneas was determined using an independent-samples *T* test and one-way ANOVA. The correlation between IOP and EC loss was determined using Pearson’s *T* test. All tests were two-tailed, and *P* < 0.05 was considered statistically significant.

## Results

### Donor corneal characteristics

In total, 942 donor cornea buttons were included in this study (Table 1). A total of 569 donors (286 males and 283 females), ranged from 0.17 to 88 years old (mean age 46.8 ± 17.7 years). A total of 373 donated both corneas, and 196 donors donated a single cornea. The most common causes of death in donors were trauma or accident in 48.2% (*n* = 274), cancer in 29.2% (*n* = 166), cardiovascular disease in 15.6% (*n* = 89), respiratory failure in 5.1% (*n* = 29), and other in 1.9% (*n* = 11). The mean death to preservation duration was 6.7 ± 3.2 h (range, 0–12 h), and the mean death to surgery duration was 5.3 ± 2.6 days (range, 1–11 days). A total of 403 donors were in-hospital patients (403/569 = 70.8%). The mean ECD was 3239 ± 440 cells/mm^2^ (range, 2016–6201 cells/mm^2^).

**Table 1:** Characteristics of the corneal donors in the virus-positive and virus-negative groups

Characteristics	Viral DNA (−)	Viral DNA (+)	Total	*P* value
Donor age (years)				*P*_*1*_ = 0.448
< 30	75 (13.7%)	2 (9.1%)	77 (13.5%)	
30–50	219 (40.0%)	12 (54.5%)	231 (40.6%)	
51–65	176 (32.2%)	6 (27.3%)	182 (32.0%)	
> 65	77 (14.1%)	2 (9.1%)	79 (13.9%)	
Donor sex				*P*_*2*_ = 0.645
Males	276 (50.5%)	10 (45.5%)	286 (50.3%)	
Females	271 (49.5%)	12 (54.5%)	283 (49.7%)	
Cause of donor death				*P_*6*_ = 0.037
Trauma or accident	270 (49.4%)	4 (18.2%)	274 (48.2%)	
Cancer	154 (28.2%)	12 (55.0%)	166 (29.2%)	
Cardiovascular disease	85 (15.5%)	4 (18.2%)	89 (15.6%)	
Respiratory failure	28 (5.1%)	1 (4.5%)	29 (5.1%)	
Other	10 (1.8%)	1 (4.5%)	11 (1.9%)	
The average time from death preservation (h)	6.7 ± 3.2	6.9 ± 2.5	6.7 ± 3.2	*P*_*4*_ = 0.782
The average time from death to surgery (d)	5.4 ± 2.6	4.5 ± 2.3	5.3 ± 2.6	*P*_*5*_ = 0.124
Inpatient status				*P_*7*_ = 0.035
Inpatient	383 (70.0%)	20 (90.9%)	403 (70.8%)	
Other	164 (30.0%)	2 (9.1%)	166 (29.2%)	
Average ECD (cell/mm^2^)	3235 ± 439	3379 ± 463	3239 ± 440	*P*_*3*_ = 0.119

*P **< 0.05**

### Virus-positive donor characteristics

Twenty-three donor cornea buttons were virus-positive according to RT-PCR (Table 1). Of the 22 cornea donors (10 males and 12 females), ranging from 0.47 to 70 years old (mean age 44.2 ± 17.1 years), the most common donor cause of death was cancer in 54.5% (*n* = 12), trauma or accident in 18.2% (*n* = 4), cardiovascular disease in 18.2% (*n* = 4), respiratory failure in 4.5% (*n* = 1), and other in 4.5% (*n* = 1). The mean death to preservation duration was 6.9 ± 2.5 h (range, 2–11 h), and the mean death to surgery duration was 4.5 ± 2.3 days (range, 1–9 days). Twenty donors were in-hospital patients (2/22 = 90.9%). The mean ECD was 3381 ± 463 cells/mm^2^ (range, 2722–4735 cells/mm^2^).

### Statistical analysis of virus-positive and virus-negative groups

Donor age, sex, ECD, time from death to the preservation, and time of death to surgery were not significantly different between the groups (*t* = 0.76, *P*_*1*_ = 0.448; χ^2^ = 0.212, *P*_*2*_ = 0.645; *t* = −1.562, *P*_*3*_ = 0.119; *t* = −0.227, *P*_*4*_ = 0.782; *t* = 1.541, *P*_*5*_ = 0.124, respectively). The cause of donor death and inpatient status was significantly different between the groups (χ^2^ = 10.235, *P*_*6*_ = 0.037 < 0.05; χ^2^ = 4.467, *P*_*7*_ = 0.035 < 0.05, respectively) (Table 1).

### Positivity rate and distribution of herpesviridae DNA

The total donor cornea herpesviridae DNA positivity rate was 2.44% (23/942). The positivity rate of CMV, HSV-1, VZV, and EBV DNA were 34.78% (8/23), 30.43% (7/23), 26.09% (6/23), and 8.7% (2/23), respectively. HSV-2 DNA was not detected in this study.

### Characteristics of recipients who received the virus-positive donor corneas

Twenty-seven recipients (4 herpesviridae-positive grafts were used in DALK and DSAEK at the same time) who received the virus-positive donor corneas were included in this study (Table 2). The ages of the 27 recipients (19 males and 8 females) ranged from 0.33 to 86 years (mean age 38.1 ± 26.0 years). The diagnoses of the recipients are listed in Table 2. Nine recipients (9/27 = 33.3%; HSV-1/CMV/VZV/EBV = 1/5/3/0) underwent penetrating keratoplasty (PK), 11 recipients (11/27 = 40.7%; HSV-1/CMV/VZV/EBV = 5/2/2/2) received corneal endothelium transplantation, and 7 recipients (7/27 = 25.9%; HSV-1/CMV/VZV/EBV = 4/2/1/0) underwent deep anterior lamellar keratoplasty (DALK).

**Table 2:** Clinical information of recipients who received the virus-positive donor corneas

NO.	Age (years)	Sex	Clinical diagnosis	Type of transplantation	BCVA	IOP (mmHg)	Virus DNA	ECD of donor (cells/mm^2^)	Last time of follow-up		ECD of graft at follow-up months postoperatively (cells/mm^2^)	
									BCVA	IOP (mmHg)	1	3
1	47	M	Pseudophakic bullous keratopathy	DSAEK	HM/10	8.0	HSVI (+)	4735	20/25	10.3	3321	3452
6	35	M	Ocular trauma	DSAEK	HM/10	30.0	HSV I (+)	3077	20/200	23.4	2711	2088
11	74	F	Fuchs endothelial dystrophy	DSAEK	20/50	11.6	HSV I (+)	2722	20/25	19.0	2548	2657
22	81	F	Bullous keratopathy due to PHACO	DMEK	20/250	10.0	HSV I (+)	*3189	20/50	15.1	2626	1800
24	54	M	Bullous keratopathy due to PHACO	DSAEK	HM/10	16.0	HSV I (+)	*3016	20/40	17.5	1695	1383
8	38	M	Alkaline burn of ocular surface	PK	HM/20	10.0	HSV I (+)	3945	HM/10 cm	12.0	3026	2526
12	25	M	Keratoconus	DALK	20/1000	6.0	HSV I (+)	–	20/20	9.9	1889	1982
21	3	F	Congenital glaucoma	DALK	LP	24.0	HSV I (+)	–	HM/10 cm	21.5	2896	3106
23	15	M	Keratoconus	DALK	FC/10	7.0	HSV I (+)	–	20/25	13.0	3414	3336
25	19	M	Keratoconus	DALK	20/1000	7.0	HSV I (+)	–	20/20	13.8	3067	3145
9	36	F	Congenital corneal leukoplakia	DSAEK	HM/40	20.0	CMV (+)	3530	20/100	18.7	2666	1387
18	22	M	Congenital corneal leukoplakia	DSAEK	HM/30	14.0	CMV (+)	3930	20/63	10.0	3008	2171
2	0.33	F	Congenital corneal leukoplakia	PK	LP	26.3	CMV (+)	3130	20/63	15.6	2945	2528
10	37	M	Thermal burn of ocular surface	PK	HM/10	9.0	CMV (+)	3764	20/40	10.2	2978	2529
13	51	M	Alkaline burn of ocular surface	PK	HM/10	6.0	CMV (+)	2788	FC/10 cm	14.5	2894	2653
17	31	M	Bullous keratopathy due to PPV	PK	HM/30	12.0	CMV (+)	3378	20/63	18.0	2392	2032
20	76	M	Alkaline burn of ocular surface	PK	HM/20	6.6	CMV (+)	3332	FC/10 cm	9.8	2930	2405
19	86	F	Scleral melting	DALK	20/50	13.0	CMV (+)	–	20/50	11.0	2306	1963
26	58	F	Corneal leukoplakia	DALK	20/200	11.0	CMV (+)	–	20/40	14.5	1839	1786
4	53	M	Prior DSAEK failure	DSAEK	20/500	22.0	VZV (+)	2986	20/63	20.3	2410	2589
14	0.42	M	Congenital endothelial dystrophy	DSAEK	LP	9.0	VZV (+)	3547	20/100	16.0	3264	3178
3	64	M	Fungal keratitis	PK	FC/10	13.0	VZV (+)	2758	20/40	18.6	2555	1529
5	0.42	M	Peter anomaly	PK	LP	26.5	VZV (+)	3545	20/63	13.4	3298	3378
7	29	M	Fungal keratitis after trauma	PK	HM/30	9.0	VZV (+)	3539	20/63	12.8	3221	3384
15	70	F	Corneal marginal degeneration	DALK	20/125	12.0	VZV (+)	–	20/100	12.0	2003	1985
16	23	M	Bullous keratopathy due to glaucoma	DSAEK	20/1000	21.0	EBV (+)	3845	20/40	20.0	3095	2639
27	0.58	M	Congenital glaucoma	DSAEK	LP	11.0	EBV (+)	3212	20/200	15.0	2893	2664

*PPV* pars plana vitrectomy, *DSAEK* Descemet’s stripping automated endothelium keratoplasty, *PK* penetrating keratoplasty, *DALK* deep anterior lamellar keratoplasty, *DMEK* Descemet’s membrane endothelial keratoplasty, *LP* light position, *HM* hand movement, *FC* finger counting, *BCVA* best-corrected visual acuity, *ECD* endothelial cell density

*The ECD of the donor the second time

### Recipient follow-up

**Fig. 1:**
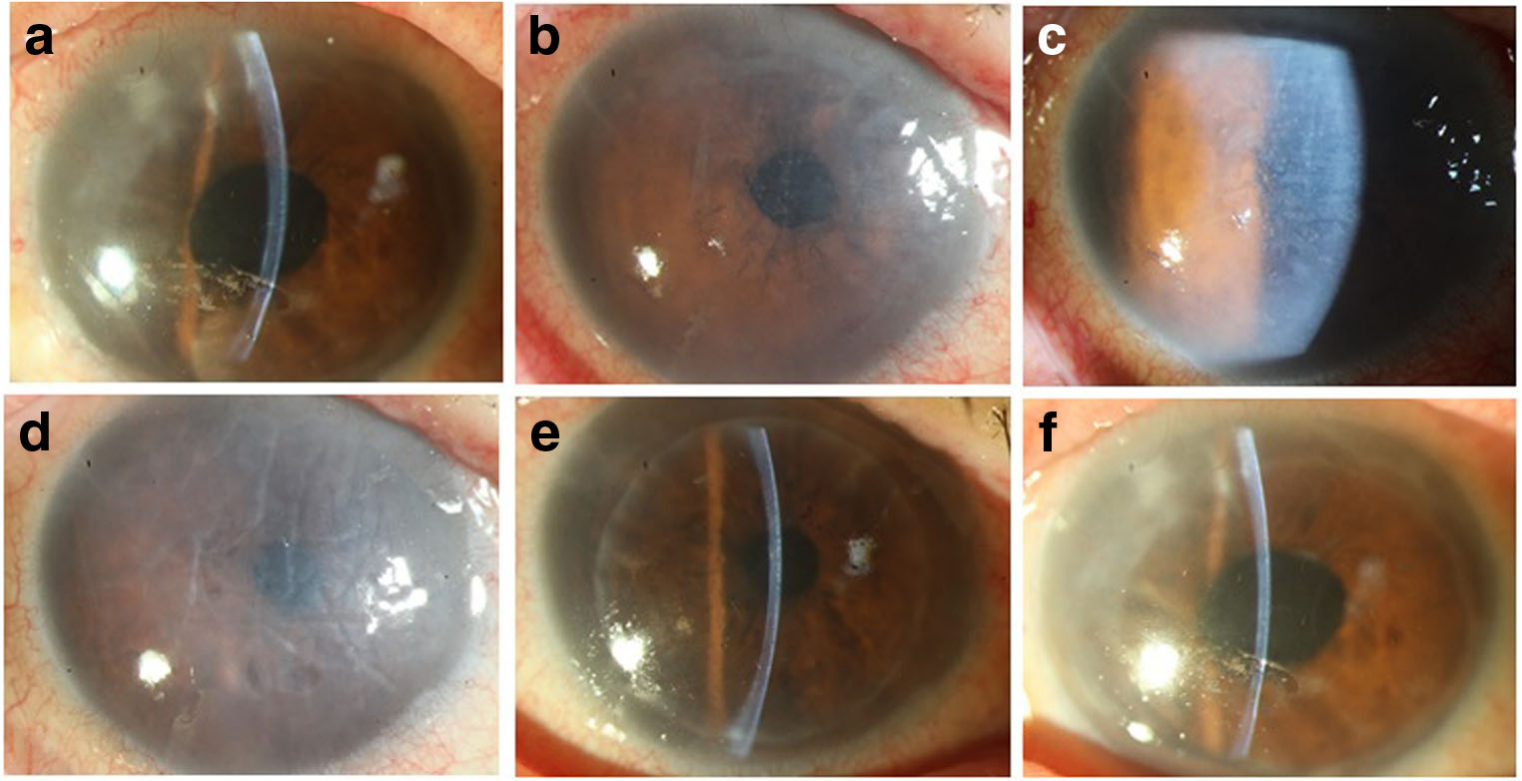
Slit lamp photographs of recipient No. 22. Preoperation (**a**), 3 days after the operation (**b**), 3 days after the operation (**c**), after intraocular injection with ganciclovir twice (**d**), 1 day after DSAEK (**e**), and 6 months after DSAEK (**f**)

**Fig. 2:**
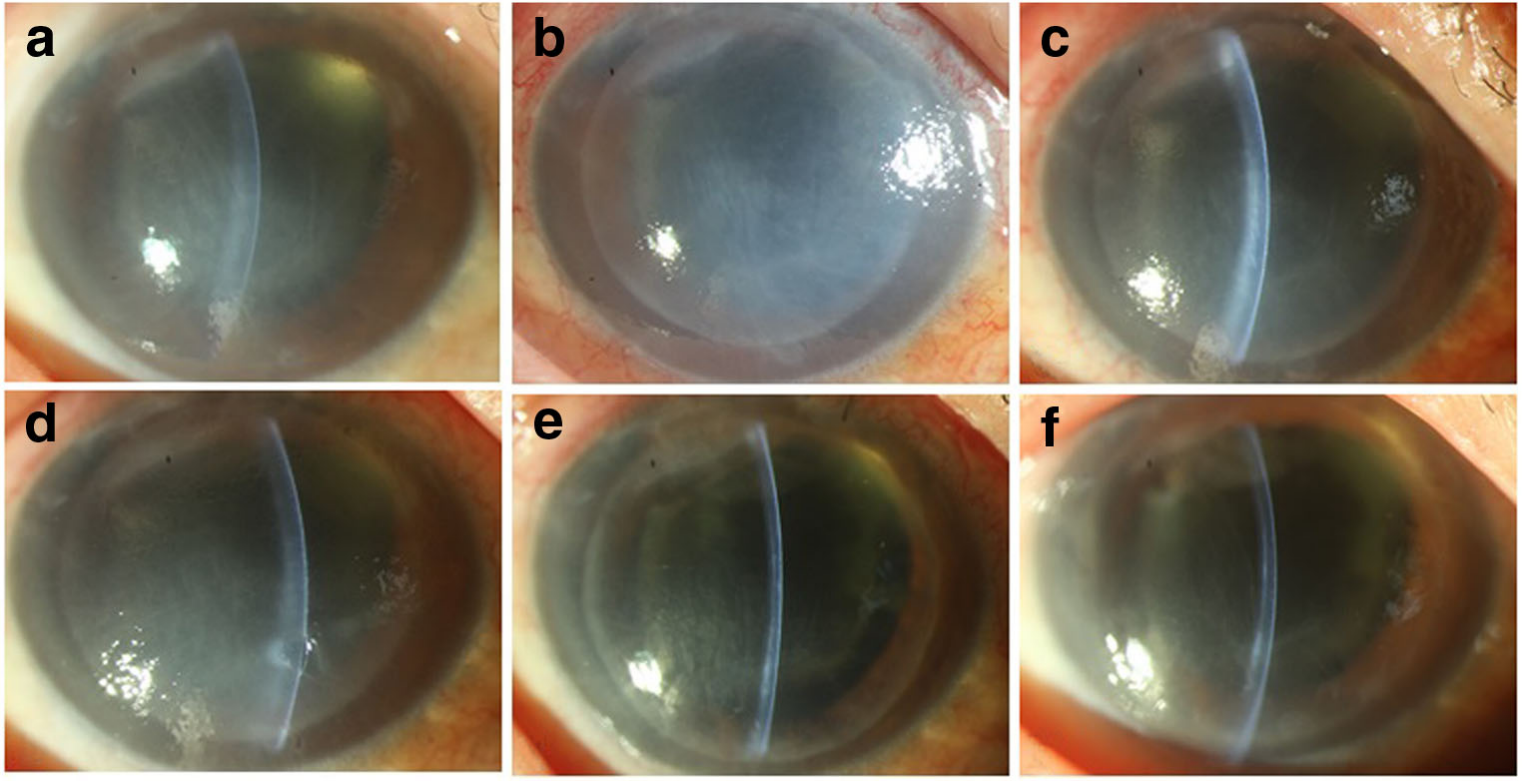
Slit lamp photographs of recipient No. 24. Preoperation (**a**), 6 days after the operation (**b**), 6 days after the operation (**c**), after intraocular injection with ganciclovir twice (**d**), 1 day after DSAEK (**e**), and 6 months after DSAEK (**f**)

The follow-up time was 3 months. AS-OCT revealed that the attachments of corneal endothelial grafts were good. Recipients Nos. 22, 23, 24, and 25 received a pair of HSV-1-positive corneas from a 50-year-old male who died in a traffic accident. Analysis of this donor’s records did not reveal any infectious diseases, and he was admitted to the intensive care unit (ICU) for 1 week before donation. Recipients Nos. 23 and 25 were both young males with keratoconus, and they underwent DALK on the same day. The transparent corneas remained completely transparent throughout the 3-month follow-up period. Recipients Nos. 22 and 24 were 81-year-old female and 54-year-old male, respectively, and both suffered from bullous keratopathy due to phacoemulsification. They underwent corneal endothelium transplantation on the same day. Recipient No. 22 showed signs of herpesviridae infection 3 days after surgery and received intraocular injections with ganciclovir twice within 4 days (aqueous humor tested HSV-1-positive) (Fig. 1b, c). Unfortunately, recipient no. 24 also showed signs of virus infection 6 days after surgery and received intraocular injection with ganciclovir twice within 14 days (aqueous humor-tested HSV-1-positive) (Fig. 2b, c). Despite an appropriate and intensive treatment course of parenteral and local therapies, the transplanted tissue did not maintain good function (Figs. 1d and 2d). It was necessary to perform Descemet’s stripping automated endothelial keratoplasty (DSAEK) half a month later (Figs. 1e and 2e). During the second surgery, the aqueous part of the removed graft and the donor corneoscleral tissue were obtained for PCR analysis; the other part of the graft was examined with transmitted electron microscopy (TEM). The aqueous and removed grafts were all positive for HSV-1 DNA, and the new donor was negative for herpesviridae DNA. TEM findings showed only denuded Descemet’s membrane (DM) without any endothelial cells left on the graft of No. 22; viral particles were spotted within the posterior stroma of the DSAEK graft of No. 24 by TEM. The grafts of recipients 22 and 24 remained transparent after retransplantation at the 6-month follow-up (Figs. 1f and 2f). This study had already been published [11].

### EC loss

The mean ECD of the grafts (except recipients who received DALK and recipients Nos. 22 and 24) was 2543 ± 561 cells/mm^2^ (*n* = 18; range, 1387–3452 cells/mm^2^) at 3 months postoperatively, representing a mean postoperative EC loss of 25.19 ± 16.25% (range, 2.39–60.71%). The EC loss in HSV-1-, CMV-, VZV-, and EBV-positive recipients was 24.40%, 32.86%, 15.57%, and 24.21%, respectively, and there was no significant difference between recipients with those viruses (*F* = 1.073, *P =* 0.392). The EC losses in those who underwent PK and DSAEK were 23.80% and 26.58%, respectively, and there were no significant differences between the two transplantation procedures (*t* = −0.344, *P* = 0.736). There was no correlation between IOP and EC loss (*P* = 0.934).

## Discussion

In total, 942 donor corneas were analyzed in our study. Twenty-three donor cornea buttons tested positive for RT-PCR, and the total herpesviridae positivity rate was 2.44% (23/942). To the best of our knowledge, this was the largest donor cornea sample number to undergo viral DNA detection among published studies, and there are no previous reports on the total virus positivity rate. After data analysis, we found that inpatient donors had a higher virus infection rate in corneal buttons and that cancer was the most common cause of death in virus-positive donors. Infections with certain viruses are strong risk factors for specific cancers. The most important infectious agents worldwide were *Helicobacter pylori*, human papillomavirus, HBV, HCV, and EBV [12]. Cancer patients with multiple organ failure are also susceptible to viral infection. Coincidentally, Broniek et al. [6] also found that hospitalized donors had an increased risk of reactivation of latent viral infections due to potential immunodeficiency resulting from severe underlying diseases or immunosuppressive therapy administered during hospitalization. Corneal eye tissue grafts obtained from donors who died in the hospital or who had cancer may have an increased risk of postsurgical endophthalmitis, possibly due to donor host microbial transmission [13]. The shortage of corneal donors has always created a bottleneck for corneal disease treatment in developing countries. We could not exclude patients who died of unknown causes or who had immunodeficiency, leukemia, or lymphoma from becoming potential organ donors, as recommended by the Eye Bank Association of America’s guidelines. For the above reasons, we suggest that the corneas from the donors who had cancer, donors who were inpatients, and donors who had immunodeficiency or who received immunosuppressive therapy should be tested for herpesviridae DNA.

The HSV-1 and HSV-2 positivity rates were 0.74% (7/942) and 0% (0/942), respectively, in our study. In a previous study, the authors mainly considered HSV. Remeijer et al. [14] found that HSV-1 DNA was detected in only 2 of 273 (0.73%) corneoscleral rims and HSV-2 was not detected. The positivity rates were the same as those in our research. However, Shimomura et al. [15] reported that HSV-1 DNA was detected in 4 of 70 (5.7%) limbal donor corneas (imported from the USA), and HSV-2 DNA was not detected in limbal corneas. We assume that the difference in the virus positivity rates might be caused by the sample sizes and donor causes of death. Quantifying HSV DNA will be our next research direction.

The positivity rates of CMV, VZV, and EBV DNA in the donor corneas in our study were 0.85% (8/942), 0.64% (6/942), and 0.21% (2/942), respectively. CMV was the most common virus detected in the donor corneas in our cohort. CMV has been increasingly implicated as a new cause of corneal endotheliitis, especially in East Asian countries [16]. Our findings also demonstrated that CMV has a higher infection rate in Asia than in our regions. VZV DNA was not detected in any of the published studies [14, 15]. A live attenuated vaccine for varicella was approved by the Food and Drug Administration and incorporated into the recommended immunization schedule for children starting in 1995 [17]. However, the VZV vaccine was not added to the schedule in China, which might explain the relatively high VZV positivity rate compared with that in other countries. Moreover, EBV is not as common as other members of herpesviridae in ophthalmological investigations, and there are no data about the EBV DNA positivity rate in the donor corneas; this might be the first report of the EBV DNA positivity rate.

The rates of EC loss in PK and DSAEK in our study were 23.80% and 26.58%, respectively, and showed no difference according to surgical method. There was no correlation between IOP and EC loss. Most recipients showed no sign of herpesviridae infection or a rejection reaction. The EC loss in HSV-1-, CMV-, VZV-, and EBV-positive recipients was 24.40%, 32.86%, 15.57%, and 24.21%, respectively, at the 3-month follow-up period. Observations of EC loss are still needed in the future to draw further conclusions. Although CMV was associated with the highest rate of EC loss, there was no evidence showing that CMV could be transmitted from graft to recipient. In our study, all 9 recipients who received CMV DNA-positive donor corneas showed no signs of CMV infection at the 3-month follow-up. Hsiao et al. [18] discovered that 4 of the 6 donor corneas containing CMV DNA failed, and only one patient was positive for CMV DNA in all three CMV endotheliitis samples during the 60.5-month follow-up period. A retrospective study conducted in London found that CMV DNA was not identified in excised failed corneal tissue or in tissue prepared for transplantation, and the authors inferred that CMV infection is not a significant risk factor for corneal graft failure in the UK [19]. The possible mechanism of CMV corneal endotheliitis might be related to anterior chamber-associated immune deviation (ACAID). The virus enters the anterior chamber, and viral particles lead to the induction of ACAID against viral antigens. Infection occurs when preexisting antibodies are incapable of neutralizing the reactivated virus. Under cell-mediated immune suppression, the virus is presumably able to proliferate efficiently in the corneal endothelium [20]. Hence, we inferred that either there was not enough CMV released into the anterior chamber to cause ACAID or that CMV was suppressed by cell-mediated immunity in recipients with normal immune function.

Several studies have reported the transmission of HSVs from corneal donors to recipients [3, 8]. Similar results were found in our research. Interestingly, 2 recipients who received HSV-1-positive corneal endothelia became infected, whereas 2 recipients who underwent DALK were not infected. Several authors have proposed that HSV may remain latent in the cornea, exhibiting nonneuronal latency [21]. Our findings might also prove that HSV remains latent in the cornea and that HSV-1 might infect the corneal endothelium before donation.

## Conclusions

In conclusion, the total virus positivity rate was 2.44% (23/942). We suggest that corneas from donors who had cancer, donors who were inpatients, and donors who had immunodeficiency or who were on immunosuppressive therapy should be tested for herpesviridae DNA in the cornea; if the eye bank does not have any conditions for herpesviridae DNA testing, transplantation of the donor corneas in immunodeficient recipients should be avoided. Moreover, the data demonstrated that HSV-1 could be transmitted from graft to recipient but that CMV could not be transmitted according to our observations. The donor corneas found to be HSV-1-positive have to be discarded and not used for keratoplasty.

## Limitations

This study was limited by its short follow-up period, which may explain why despite the positive herpesviridae DNA results, the patients showed no symptoms of infection after transplantation. Therefore, we plan to continue observing patients with positive DNA results. Additionally, the minimum RT-PCR limit of detection might have caused false-negative results in our study; RT-PCR can only detect the presence of viral DNA and the activity of the virus is still unknown.

## Data Availability

Not applicable.
